# Ultra-high-resolution quanta image sensor with reliable photon-number-resolving and high dynamic range capabilities

**DOI:** 10.1038/s41598-022-17952-z

**Published:** 2022-08-16

**Authors:** Jiaju Ma, Dexue Zhang, Dakota Robledo, Leo Anzagira, Saleh Masoodian

**Affiliations:** Gigajot Technology, Inc., Pasadena, CA USA

**Keywords:** Engineering, Electrical and electronic engineering

## Abstract

Superior low-light and high dynamic range (HDR) imaging performance with ultra-high pixel resolution are widely sought after in the imaging world. The quanta image sensor (QIS) concept was proposed in 2005 as the next paradigm in solid-state image sensors after charge coupled devices (CCD) and complementary metal oxide semiconductor (CMOS) active pixel sensors. This next-generation image sensor would contain hundreds of millions to billions of small pixels with photon-number-resolving and HDR capabilities, providing superior imaging performance over CCD and conventional CMOS sensors. In this article, we present a 163 megapixel QIS that enables both reliable photon-number-resolving and high dynamic range imaging in a single device. This is the highest pixel resolution ever reported among low-noise image sensors with photon-number-resolving capability. This QIS was fabricated with a standard, state-of-the-art CMOS process with 2-layer wafer stacking and backside illumination. Reliable photon-number-resolving is demonstrated with an average read noise of 0.35 e- rms at room temperature operation, enabling industry leading low-light imaging performance. Additionally, a dynamic range of 95 dB is realized due to the extremely low noise floor and an extended full-well capacity of 20k e-. The design, operating principles, experimental results, and imaging performance of this QIS device are discussed.

## Introduction

With the steady advancement of CMOS fabrication processes, small pixels with 1 μm pitch size or less are now possible, which allows for a high-density integration of 10–100 s of mega-pixels (MP) in a limited silicon area^1–3^. Driven by smartphone imaging applications in recent years, these ultra-high-resolution image sensors have brought new capabilities like flexible digital zooming, cropping, and down-sampling to digital photography. Furthermore, combining these sensors with machine learning based image reconstruction methods makes image detail enhancement, noise reduction, and pattern recognition possible. These features are also widely desired in cinematography, industrial imaging, medical diagnostics, and scientific imaging such as large-field-of-view microscopy with dynamic observation of live cells. However, with small pixel sizes, standard CMOS image sensors often face performance limitations in both low-light and high-dynamic-range (HDR) situations. The reduced photon flux per pixel and the smaller pixel photoelectron storage capacity, often referred to as the full-well capacity (FWC), means these sensors suffer from poor low-light signal-to-noise ratio (SNR) and saturate too quickly in bright light conditions. To overcome these limitations, the QIS concept of an array with hundreds of mega-pixels to giga-pixel resolution of specialized small pixels that are capable of photon-number-resolving, high-speed readout, and HDR, was proposed^4,5^. With a QIS, the low-light imaging performance is greatly enhanced due to the photon-number-resolving capability despite the small pixel sizes, and the dynamic range is improved because of the extended sensitivity in the low-light regime. The development of a QIS-based detector using an existing CMOS active pixel platform started from 2012 at Dartmouth College. To realize a photon-number-resolving pixel, the total input-referred readout noise (read noise) was reduced by lowering the capacitance of the sense node, referred to as floating diffusion (FD), with novel pixel device structures^6^. In this case, the voltage signal generated by every photoelectron, referred to as conversion gain (CG), is greatly increased to easily overcome the background noise from the rest of the readout circuits in the sensor. A photon-number-resolving active CMOS pixel was first demonstrated in 2015 with a sub-0.4 e- read noise. This breakthrough has matured over the last 5 years and has been implemented into 1MP, 4MP, and 16MP QIS devices^7–9^. These new QIS devices were an expansion from the original proposed single-bit QIS concept, and they contain arrays with detectors that both have reliable multi-bit photon-number-resolving and HDR capabilities. The high CG pixels in these devices have a relatively large full-well capacity with the inclusion of a dual pixel gain (DPG) structure in a 4MP array size to simultaneously enable photon-number-resolving and HDR imaging^8^.

Around the same time, QIS detectors based on single photon avalanche diodes (SPAD) were also being actively developed. SPADs use electron avalanche multiplication to realize ultra-low read noise when one or more photoelectron are detected. Different from the CMOS active pixels based QIS, SPADs can detect the single-photon events but cannot resolve the number of photoelectrons: the binary outputs of SPADs will be saturated when there are more than one photoelectrons detected in a single event. This introduces additional non-linearity to the output signal that is not desirable in the scientific applications where the exact photon counts are sought for. In the state-of-the-art SPADs, the single photon events in each pixel are recorded with a pixel-level analog/digital counter^10–15^, such that the total counts in each pixel reflect the intensity of the incident light at each location. The power consumption and pixel size of the SPAD sensors with per-pixel digital counter is proportional to the counter bit depth and speed, effectively limiting the further shrink of the pixel size and the improvements of pixel resolution and dynamic range. However, different design techniques, including multiple-exposure with time-gating and extrapolation are being explored to overcome this hurdle^16,17^. Additionally, the further shrink of the pixel size of SPAD sensors is also limited by the complexity of the pixel-level readout circuits and the physical area required for the inter-pixel isolation^18^, which makes the further shrink of the pixel pitch size to 1 μm level extremely unlikely in the near future. The SPAD sensor with the highest pixel resolution reported so far is a 3.2MP device with a 7.6 μm pixel size^15^. These current limitations have made CMOS active pixel-based QIS more desirable for realizing ultra-high resolution image sensors with photon-number-resolving and HDR capabilities for use in smartphones, cinematography, industrial imaging, medical diagnostics, microscopy and other imaging applications. In this paper, a CMOS active pixel-based QIS with 163MP resolution and 1.1 μm pixel pitch size and 1.26-inch optical format is reported. This is the highest pixel resolution ever reported among QIS detectors and photon-number-resolving sensors in general. This ultra-high resolution QIS device supports both 163MP and 41MP operation without any noise increase due to the in-pixel 2 × 2 charge binning, and the dual pixel gain readout realizes 0.35 e- rms read noise and 95 dB single-exposure dynamic range simultaneously at room temperature operation.

## Results

### Sensor design and operating principles

**Figure 1:**
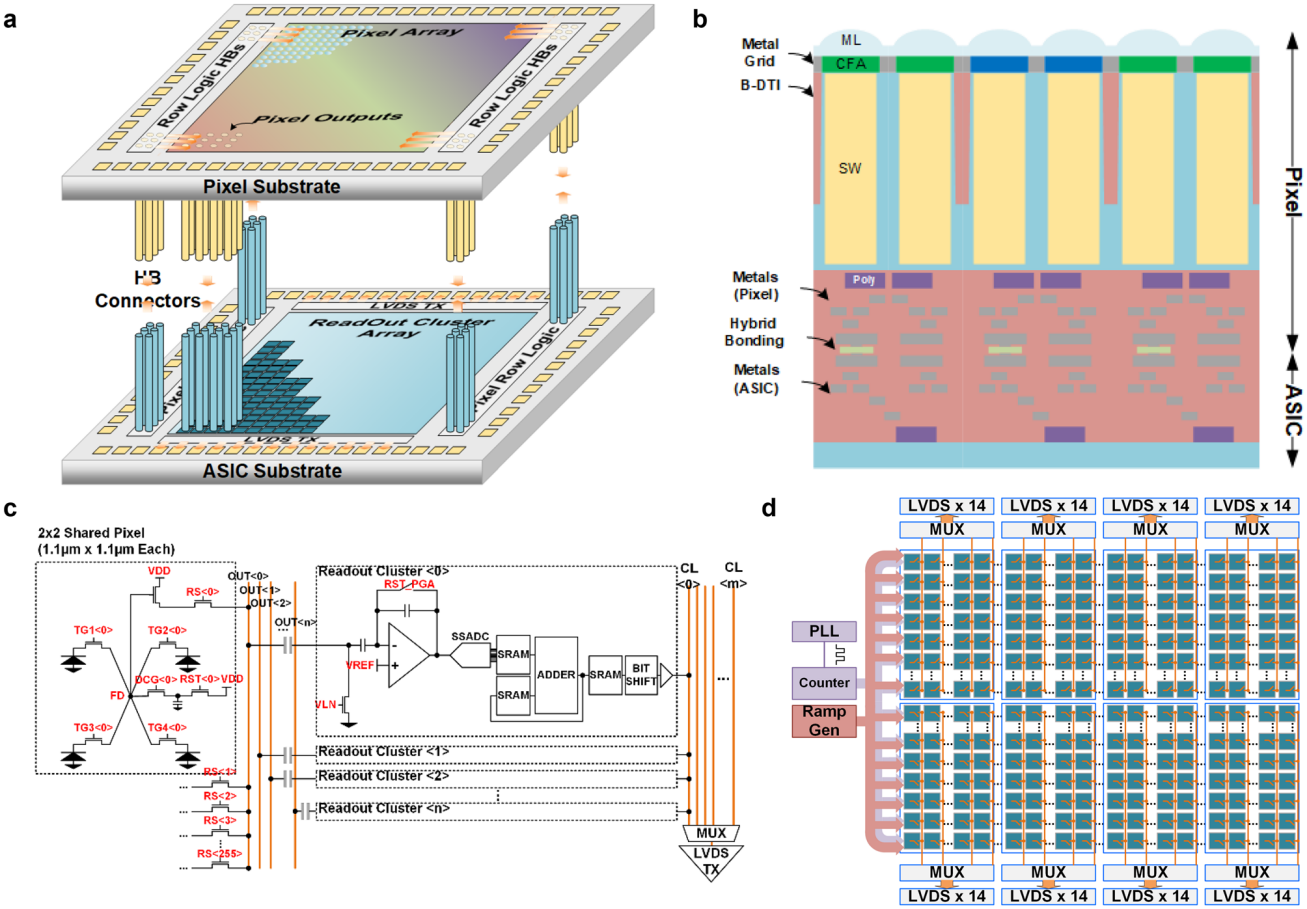
Illustrations of the architecture and the QIS. (**a**) Illustration of the structure of the wafer-to-wafer hybrid bonding connections. (**b**) Illustration of the cross-section of the sensor. (**c**) Schematic of the pixel clusters and the readout circuit clusters on the ASIC substrate. (**d**) Readout scheme and architecture of the readout clusters on the ASIC substrate.

This sensor was designed and fabricated at Taiwan semiconductor manufacturing company (TSMC) with a 45 nm/65 nm stacked BSI CMOS image sensor process^19,20^. The sensor architecture is shown in Fig. 1. As shown in Fig. 1a, the QIS was designed with a 2-layer stacking structure, where the pixels are located on the top wafer (pixel substrate) and the readout circuits are located on the bottom wafer (ASIC substrate). The pixel array contains a 14,464 (H) × 11,264 (V) array of 1.1 µm-pitch pixels. The control signals for the pixels are sent from the ASIC substrate to the pixel substrate through high-density wafer-to-wafer hybrid bonding (HB) connectors located on the left and right sides of the pixel array^21,22^. The pixel output signals are connected to the ASIC substrate through HBs located within the pixel array. The cross-section of the sensor structure is shown in Fig. 1b. The optical structures are built on the backside of pixel substrate and include the micro-lenses (ML), the color-filter array (CFA), and a metal grid. To enable 2 × 2 pixel binning, this sensor uses a Quad Bayer CFA structure, where every 2 × 2 pixel unit shares the same color filter. The backside deep trench isolation (B-DTI) is formed for every 2 × 2 pixels to reduce the optical and electrical cross-talk between different colors. Each 1.1 μm-pitch pixel in the 2 × 2 group has an independent photodiode storage well (SW).

The readout circuitry on this QIS device uses a cluster-parallel architecture^23^. The large pixel array and the array of readout units are divided into much smaller sub-arrays, referred to as clusters. As shown in Fig. 1c, each cluster of pixels is connected to a dedicated readout cluster located on the ASIC substrate through HB connectors. The 163MP array is divided into 452 (H) × 352 (V) clusters, with each cluster consisting of 2 (H) × 512 (V) pixels. The area of the readout cluster is 35.2 µm × 35.2 µm to match the area of the pixel cluster of 2.2 µm × 563.2 µm. The readout cluster contains a pixel source follower biasing unit (VLN), a programmable gain amplifier (PGA) with 1 V/V to 8 V/V programmable analog gain, a single-slope analog-to-digital converter (SSADC) with a programmable bit depth from 1 to 14bit, and a programmable digital correlated multiple sampling (CMS) block that consists of an adder and a bit-shifter. As shown in Fig. 1d, a phase locked loop (PLL) and a ramp generator are also included as peripheral circuitry on the ASIC substrate. The PLL generates a 1.6 GHz clock signal that drives a high-speed, gray-coded counter with programmable bit depth from 1 to 14bit. The high-speed counter outputs and the ramp output are uniformly distributed to the SSADCs in the 452 (H) × 352(V) clusters. Because of the large number of readout clusters and the large sensor area, the loading for the ramp and counter signals is heavy. To ensure the uniformity and integrity the signals, the signals are enhanced with sub-drivers placed inside the cluster array between clusters. The cluster-parallel architecture allows all of the clusters to function in parallel. In each readout cycle, the data from 159,104 pixels are read out simultaneously, so 2 × 512 (1024 pixels per cluster) cycles are needed to readout the entire array. The data from the readout clusters are sent off-chip through high-speed LVDS interface with 66 (14 × 4) lanes located on both the top and bottom of the sensor. Each LVDS lane can operate at a max speed of 400MSPS, which supports a maximum frame rate of 7.5 fps at 163 Mpix and 30 fps at 41 Mpix resolution with 2 × 2 pixel binning both outputting 28bits per pixel (14bit reset and 14bit signal). As shown in Fig. 1d, the readout clusters are divided into 8 sections, and all the clusters in each section are multiplexed into their respective 14 LVDS output lanes. In each readout cycle, the clusters in each section are selected sequentially for the data readout.

The pixels use a 2 × 2 shared readout^24^ with a DPG structure that supports an intra-scene switchable conversion gain for single-exposure HDR imaging. The parasitic capacitance on the FD is minimized using the pump-gate structure and an optimized pixel layout^6^ to improve CG in the high CG (HCG) readout mode. Additionally, a buried-channel source follower^25^ was used to reduce the 1/f noise and random telegraph noise (RTN). An additional in-pixel capacitor can be connected to the FD to lower the conversion gain in the low CG (LCG) mode to allow more charge to be transferred to the FD without saturating and to increase the FWC. This mode is only needed when the 2 × 2 charge binning is enabled, as the FD does not have enough voltage swing to sense all of the photoelectrons of a filled 2 × 2 photodiode group. A row-wise DCG signal is used to switch between the HCG and the LCG modes during readout.

**Figure 2:**
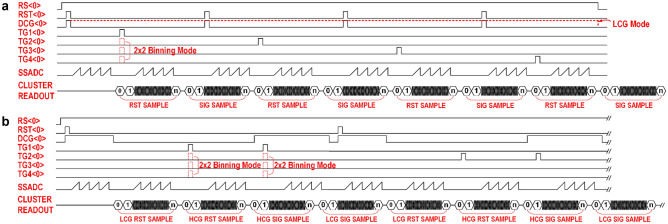
Illustrations of the operating timing of the QIS. (**a**) Simplified sensor operating timing diagram for the single-pixel-gain readout mode. In this example, CMS-4 readout is being used. (**b**) Simplified sensor operating timing diagram for the dual-pixel-gain readout mode. In this example, CMS-4 readout is being used.

The typical timing for operating this QIS under the single-gain mode and the DPG mode are shown in Fig. 2a and b, respectively. To start the readout operation, the reset gate (RST) and DCG signals are both pulsed to reset the voltage on the FD in each pixel. For the HCG readout, the DCG signal remains low after the FD reset is completed, and for the LCG readout, the DCG signal remains at the high level after the FD reset to connect the FD with the in-pixel capacitor. Correlated double sampling (CDS)^26^ or programmable correlated multiple sampling (CMS) are performed in the digital domain. First, the FD reset level is sampled by the SSADC between one and eight times depending on the programmed CMS number. All of the conversion samples are added by the digital adder and averaged by the bit-shifter. This multiple sampling operation is used to further suppress the readout noise incurred by the readout signal chain. The final output of the pixel reset level is sent off-chip after all of the ADC operations are complete, and all the clusters have been selected for data readout. Next, the transfer gate (TG) is pulsed to move the accumulated photoelectrons in the pixel SW to the FD to induce a voltage change proportional to the number of collected photoelectrons. When the 2 × 2 binning mode is enabled, the four TGs are pulsed simultaneously. After the charge is transferred, the new FD voltage is sampled again up to eight times by the SSADC and read out off-chip. The reset and signal levels are then subtracted off-chip to complete the correlated sampling process. When the DPG readout is used, each pixel produces four outputs: LCG reset and signal, as well as HCG reset and signal. The two correlated pairs of outputs are subtracted off-chip, and pixel-wise processing is performed to select between the LCG and HCG output for HDR image reconstruction.

### Experimental results

**Figure 3:**
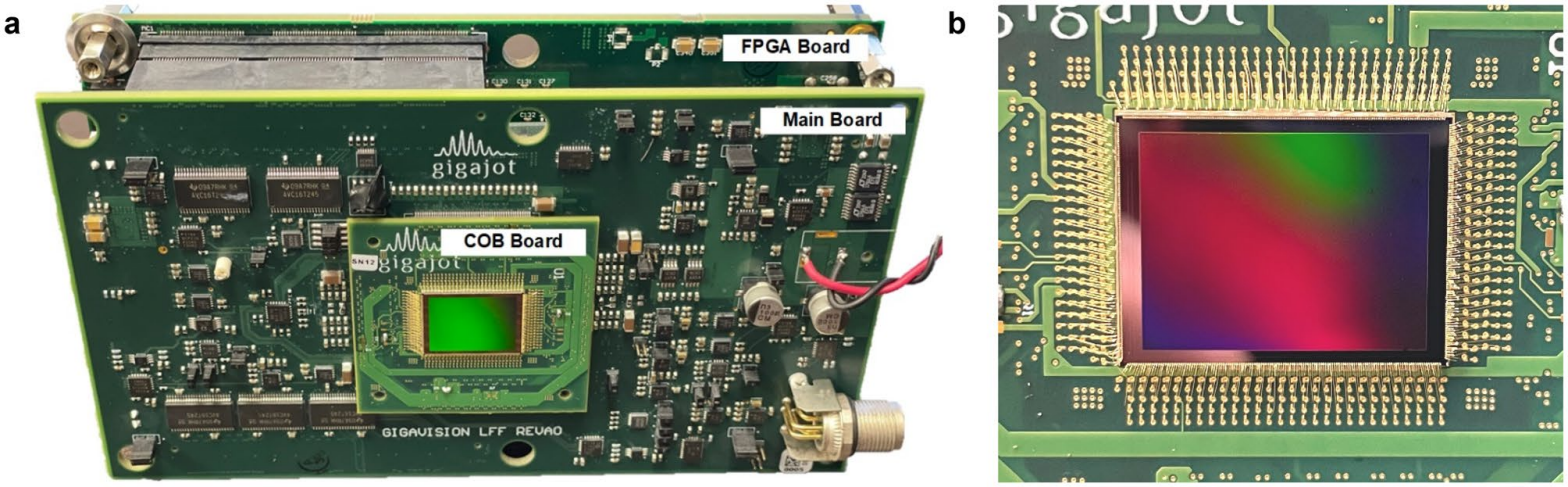
(**a**) The test system developed for the evaluation of the sensor that includes a main PCB, a COB PCB, and a FPGA PCB. (**b**) The photo of the sensor on the COB board shot from the backside of the pixel array (illumination side).

The test system developed for the evaluation of this QIS-based detector is shown in Fig. 3a. This system involves 3 printed circuit boards (PCB): a chip-on-board (COB) PCB, a main PCB, and a field programmable gate array (FPGA) PCB. The COB PCB shown in Fig. 3b serves as the wire-bonding platform to connect the 498 pads on the sensor to the test system. The COB PCB is connected to the main PCB via four board-to-board connectors. The main PCB contains multiple low dropout (LDO) voltage regulators and current sources to provide the power supplies and reference currents for the QIS. The FPGA PCB contains a Xilinx Kintex Ultrascale FPGA device that provides the sensor control signals, captures the sensor output data, temporarily stores the sensor data in local memory, and sends the sensor data to a computer through a USB3 interface.

**Figure 4:**
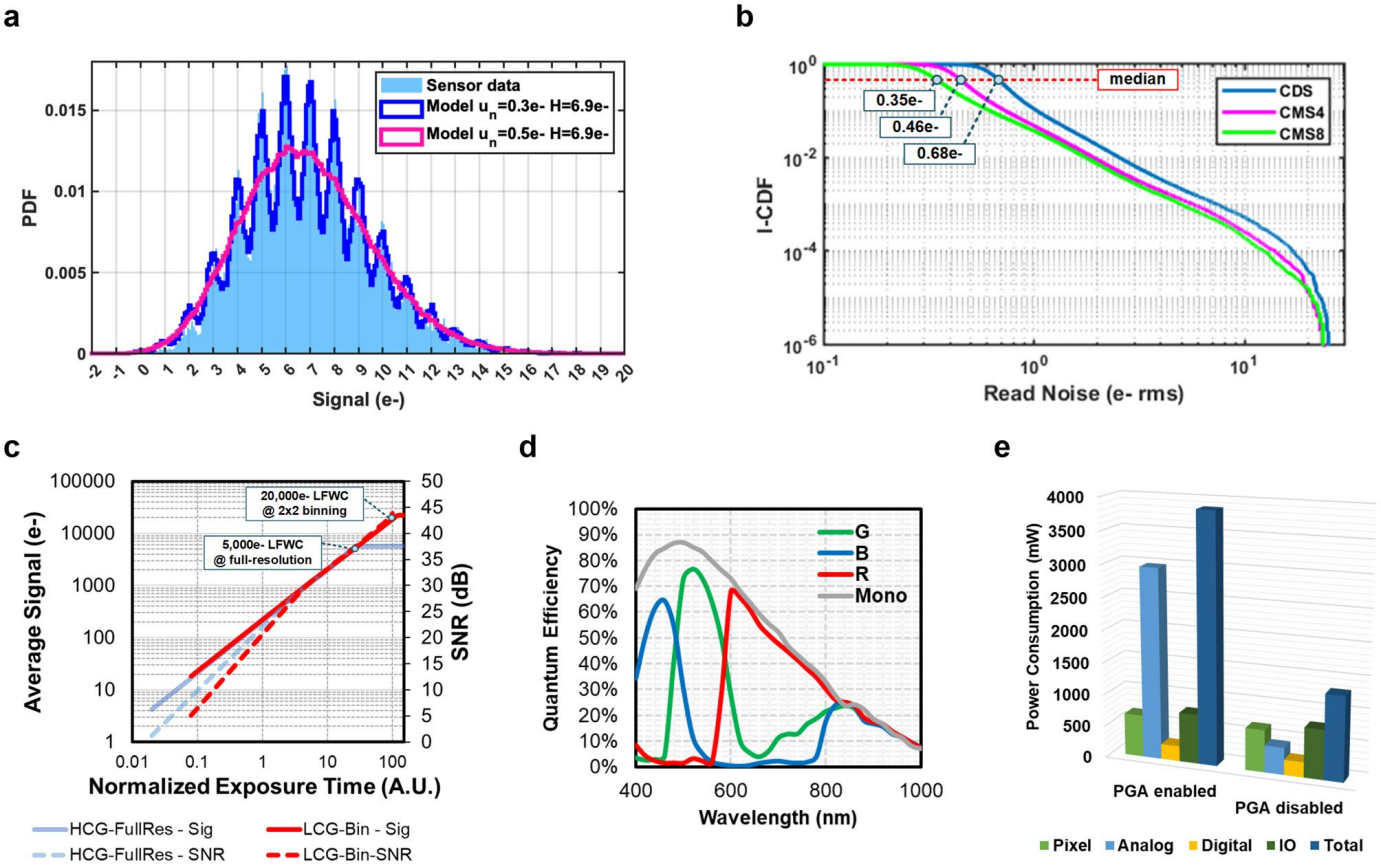
Key sensor performance. (**a**) Photon counting histogram formed with the data from one pixel in 12,000 frames. The sensor data is well aligned with the distribution predicted by the theoretical Poisson-Gaussian model with 6.9e- average signal and 0.3e- read noise. The results are also compared with a model response of a sensor with 0.5e- read noise (e.g., sCMOS sensor), and the model shows no distinctive photon quantization or photon number resolving capability. (**b**) Inverse cumulative density function (I-CDF) of read noise distribution across the pixel array with CDS, CMS4, and CMS8 readout. (**c**) Average signal and signal-to-noise ratio vs. normalized exposure time under HCG and LCG modes with full resolution and 2 × 2 binning. (**d**) Quantum efficiency curves of the sensor in RGB channels from 400 to 1000 nm. (**e**) Distribution of power consumption in different domains on the sensor with PGA enabled and disabled.

The photon-number-resolving capability of individual pixels in the array is demonstrated with the photon-counting histogram (PCH) method^27^. For a sensor with < 0.5 e- rms read noise, the quantization of photoelectrons can be observed in a pixel’s output data histogram so photon-number-resolving can be realized. This model describes the output of a photodetector using a Poisson-Gaussian distribution, where the Poisson distribution models the randomness of discrete photon arrivals, and the Gaussian distribution models the continuous voltage fluctuation of the output signal introduced by the read noise. The probability density function (PDF) of the Poisson-Gaussian distribution is given by:

1 $$P\left( U \right) = \mathop \sum \limits_{k = 0}^{\infty } \left\{ {\frac{1}{{\sqrt {2\pi u_{n}^{2} } }}\exp \left[ { - \frac{{\left( {U - k} \right)^{2} }}{{2u_{n}^{2} }}} \right]\frac{{e^{H} H^{k} }}{k!}} \right\}$$

where $k$ is the discrete number of photoelectrons, $u_{n}$ is the input-referred read noise, and $H$ is the average number of photoelectrons accumulated in the detector per integration period. A PCH generated from experimental sensor data is shown in Fig. 4a. This PCH is formed with 12,000 samples from one pixel under a stable illumination, using CMS8 readout, and a 25 °C junction temperature. The experimental data shows good alignment with the theoretical model using $H=6.9$ e- and ${u}_{n}=0.3$ e- rms. However, the read noise varies across the entire pixel array due to the unavoidable fluctuations and variability in the fabrication process. The statistical read noise distribution of the entire pixel array was measured using consecutive image frames taken under dark conditions with a junction temperature of 25 °C. The read noise with different CMS cycles was compared and the inverse cumulative density function (I-CDF) curves of the read noise distributions are shown in Fig. 4b. The median read noise is 0.35 e-, 0.46 e-, and 0.68 e- with CMS8, CMS4, and CDS (CMS1), respectively. With CMS8, more than 80% of the pixels satisfy the minimum requirements for photon-number resolving with a read noise < 0.5 e- rms^28,29^ and ~ 97% of the pixels have < 1 e- read noise under room temperature. The pixels with higher read noise are often associated with the RTN from the in-pixel source followers induced by interface traps^30^. In addition to the ultra-low read noise, extremely low fixed pattern noise (FPN) and dark current were also demonstrated. The measured average dark current under 60 °C is 1.64 e-/sec/pixel at full resolution and 7.60 e-/sec/pix using 2 × 2 binning. The measured total dark FPN is 0.2e- rms both with and without the CMS noise reduction. Similar to the column FPN that often presents in the sensors with the traditional column-parallel readout, cluster-wise FPN in cluster-parallel readout architecture can be generated by the small fabrication variations in each readout clusters. In our measurements, the cluster-wise FPN is as low as 0.03 e- rms. Such low FPN level can be realized thanks to the significantly relaxed area aspect ratio for the analog layout design in the cluster-parallel architecture.

The photo-response characteristics of the sensor was measured with a tunable color temperature LED light source, so that the response from the 3 color channels could be balanced. A photon transfer curve (PTC) measurement was used to characterize the signal response of the sensor, where the light source was kept at a stable photon flux level and color temperature while the exposure time of the sensor was swept from the shortest (~ 100 μsec) to ~ 10 ms. As shown in Fig. 4c, the sensor shows a linear response versus the exposure time up until the output is saturated. The measured linear FWC is 5000 e- at full-resolution and 20,000 e- with 2 × 2 pixel binning. Note that the linear FWC is the same for the HCG and LCG modes when the sensor is operating at full resolution, because the linear FWC is limited by the capacity of the photodiode instead of the voltage swing of the FD. When 2 × 2 binning is enabled, the measured linear FWC is ~ 6000 e- with the HCG readout, due to the limits on the voltage swing of the FD. Hence, the LCG mode is required to read out the maximum capacity of 20,000 e- from the 2 × 2 photodiodes. The resulting maximum SNR at full resolution and 2 × 2 binning are 37 dB and 43 dB, respectively. Within the linear output range, the sensor shows good linearity with < 0.5% of non-linearity. Finally, low photo-response non-uniformity (PRNU) of 1.0% at full-resolution and 0.8% with 2 × 2 binning was also demonstrated.

The sensor also demonstrated high quantum efficiency (QE) in the visible wavelength region. The QE was measured with a National Institute of Standards and Technology (NIST) calibrated light power meter and a monochromator that generates light with wavelengths from 400 to 1000 nm and a narrow bandwidth of 10 nm. The QE curves for the monochrome and color versions of the sensor are shown in Fig. 4d. The monochrome sensor has a peak QE of 87% at 480 nm and a QE between 10 and 20% in the near-infrared (NIR) region. The color sensor has a peak QE of 77% at 520 nm in the green channel.

2 $${\text{FOM}} = {\text{power}}\;[{\text{W}}]\; \times \;{\text{read}}\;{\text{noise}}\;[{\text{e - rms}}]/\{ {\text{num}}.\;{\text{of}}\;{\text{pixels}}\; \times \;{\text{frame}}\;{\text{rate}}\;[{\text{fps}}]\; \times \;2^{ \wedge } \;({\text{ADC resolution}})\}$$

The distribution of the sensor power consumption is shown in Fig. 4e. The power consumption was measured with 14bit ADC resolution, full pixel resolution, and 7.5 fps frame rate. The total power consumption of the sensor is 3.86 W with the PGA enabled and 1.32 W with the PGA disabled. The large number of readout clusters on the sensor (~ 160 k) each include 4 PGAs which consume a significant amount of power in total (~ 2.5 W). The median read noise is 0.85 e- rms with CMS8 readout and the PGA disabled, which would be sufficient for many applications where power consumption may be a concern. The figure of merit (FOM) in Eq. 2 is used to evaluate the power efficiency while taking into account the low-noise performance, frame rate and array size for comparison with other sensors. The FOM of this sensor is 0.067 e-·pJ/pixel/LSB with the PGA enabled and 0.056 e-·pJ/pixel/LSB with the PGA disabled. This power efficiency is very competitive when compared to other state-of-the-art cellphone CIS. The overall performance of the reported sensor is compared with other state-of-the-art high-resolution and low-noise image sensors in Table 1. Compared with the cellphone CIS, the reported sensor shows distinctive advantages in the low-light imaging related performance including read noise and dark current as well as the low-noise power efficiency. Compared with other high-sensitivity image sensors such as scientific CMOS (sCMOS) image sensors^31^ and electron-multiplication charge coupled devices (EMCCDs)^32^, the reported sensor demonstrates a significant improvement of the pixel resolution with similar optical format, while realizing much lower read noise and dark current to realize reliable photon-number-resolving capability.

**Table 1:** Performance summary and comparison with the state-of-the-art image sensors.

Parameter	This work	State-of-the-art cellphone CIS^1,2^	State-of-the-art sCMOS^31^	State-of-the-art EMCCD^32^
Manufacturing process	45 nm/65 nm stacked BSI	40 nm/22 nm stacked BSI		
Pixel resolution	14,464 (H) × 11,264 (V) 163MP	16,384 (H) × 12,288 (V) 200MP	4096 (H) × 2300 (V) 9MP	1024 (H) × 1024 (V) 1MP
Pixel pitch size	1.1 µm	0.612 µm	4.6 µm	13 µm
Pixel architecture	2 × 2 shared readout with DPG	4 × 2 shared readout		
Full-well capacity	5000 e- @ full-resolution 20,000 e- @ 2 × 2 binning	5000 e-	7000 e-	80,000 e-
Read noise	**0.35 e- rms** **@ 8 V/V analog-gain, CMS8 ** **0.85 e- rms** **@ 1 V/V analog-gain, CMS8**	1.60 e- rms @ 16 V/V analog-gain	0.5 e- rms @ 32 V/V analog gain	< 1 e-@1000 V/V gain
Single-exposure dynamic range	**83 dB @ full-resolution** ** 95 dB @ 2 × 2 binning and DPS**	70 dB	83 dB	
Peak QE	87%		> 85%	95%
Dark current density @ 60 °C	**1.35 e-/sec/µm**^**2**^	3.47 e-/sec/µm^2^	3.02 e-/sec/µm^2^ *(estimated from dark current at 30 °C and 6 °C doubling temperature)*	156.30 e-/sec/µm^2^ *(estimated from dark current at 20 °C and 6 °C doubling temperature)*
Max frame rate	7.5 fps @ 163MP 30 fps @ 2 × 2 binning, 41 MP	8 fps @ 200MP 24 fps @ 50MP	120 fps@9MP	20 fps @ 1MP
Power consumption	3.86 W (30 fps, 14bit, w/PGA) 1.32 W (30 fps, 14bit, w/o PGA)	1.25 W(maxfps, 10bit)	1.8 W(max fps, 11bit)	
FOM (Eq. (2))	**0.067 e-pJ/pixel/LSB (30 fps, 14bit, w/PGA) ** **0.056 e-pJ/pixel/LSB (30 fps, 14bit, w/o PGA)**	1.21 e-pJ/pixel/LSB (8 fps, 10bit)	0.39 e-pJ/pixel/LSB (120 fps, 11bit)	

Significant values are in bold.

**Figure 5:**
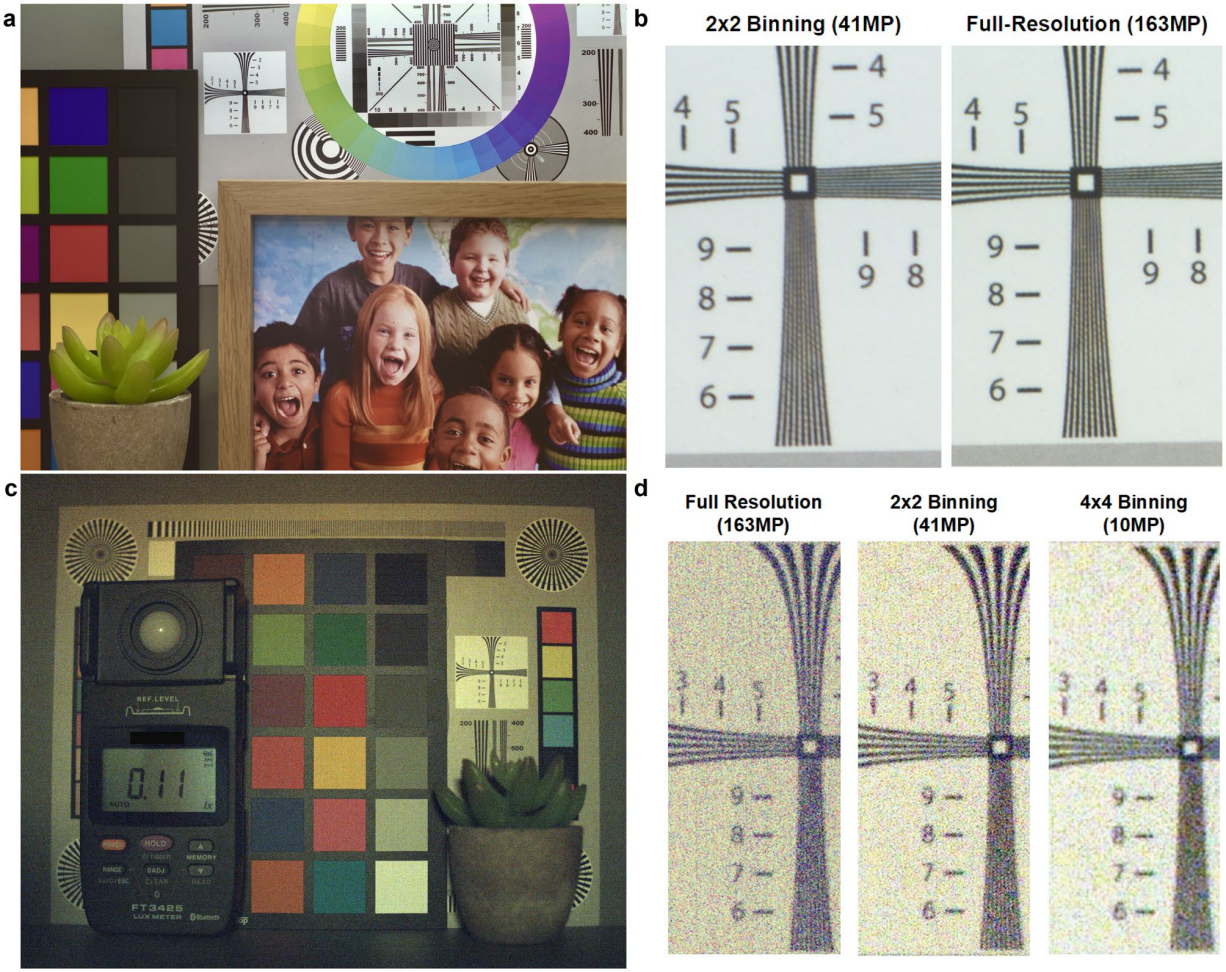
Sample images under office lighting condition (~ 400 lx) and ultra-low-light condition (110mlux). The high-light images were taken with F/4 lens and 40 ms exposure time, and the low-light images were taken with F/1.4 lens and 160 ms exposure time. (**a**) Full-resolution sample image under ~ 400 lx with demosaicing, white-balance, color correction matrix, and gamma correction applied. (**b**) Comparison of spatial resolution with 2 × 2 binning and full resolution. (**c**) 2 × 2 binning sample image under 110mlux with demosaicing, white-balance, color correction matrix, and gamma correction applied. (**d**) Comparison of spatial resolution with full resolution, 2 × 2 binning and 4 × 4 binning (2 × 2 charge binning + 2 × 2 digital binning).

The imaging performance of the QIS device was tested under different lighting conditions. A sample image under office lighting conditions (~ 400 lx) is shown in Fig. 5. This sample image was taken with a F/4.0 lens and 40 ms exposure time. The image was re-constructed from the raw sensor outputs, and only standard image processing blocks such as demosaicing, white-balance, color correction matrix, and gamma correction were applied. For the full-resolution image, a customized demosaicing algorithm for a Quad Bayer CFA with spatial interpolation is used. The spatial resolution of the full-resolution (163MP) image is compared to the 2 × 2 binned image (41MP) in Fig. 5b. A higher line resolution can clearly be observed in the full-resolution mode.

Sample images taken under ultra-low-light condition are shown in Fig. 5c and d. These images were taken under 110 mlux with a F/1.4 lens, 160 ms exposure time, and different pixel binning options. An image with 2 × 2 charge-binning is shown in Fig. 5c. Only standard image processing block including demosaicing, white-balance, color correction, and gamma correction are applied to this image. The ultra-low read noise, dark current, and high QE make this QIS capable of capturing high-quality full-color images under these very low-light conditions. The zoomed-in images in Fig. 5d compare the spatial resolution of the full resolution image, the 2 × 2 charge binning image, and a 4 × 4 binning (2 × 2 charge binning + 2 × 2 digital binning) image. The SNR in the images increases with the binning ratio although this typically comes at the expense of spatial resolution. However, in this case the full resolution image does not show noticeably better spatial resolution because of the relatively low SNR seen by the small pixel size with this scene.

**Figure 6:**
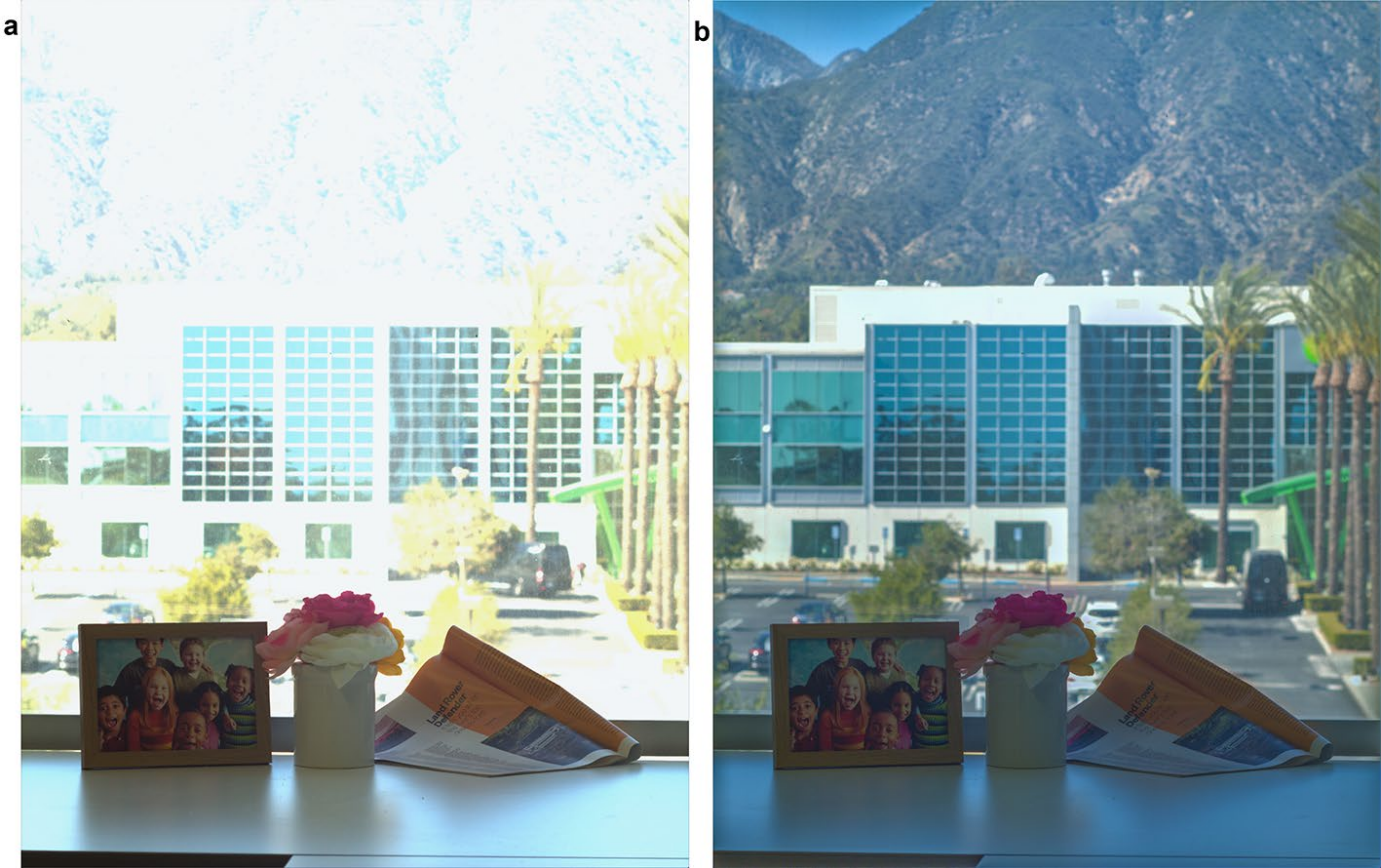
Sample images under HDR condition with and without DPG operation. The sample images were taken 2 × 2 binning with a F/8.0 lens and 100 ms exposure time. (**a**) HCG only. (**b**) DPG mode.

The HDR performance of the QIS is shown in Fig. 6. The sample images were taken using 2 × 2 charge binning, a F/8.0 lens and a 100 ms exposure time. Both indoor and outdoor scenes are included in this single image. When the DPG is not enabled, the FWC is ~ 6000 e- and the pixels imaging the outdoor portion of the scene are saturated because of the limited dynamic range. With the extended FWC of 20,000 e- in the DPG mode, both indoor and outdoor scenes can be well represented in a single image with little loss of detail (Fig. 6b).

## Discussion

In this paper, we present a 163MP ultra-high-resolution QIS that simultaneously enables photon-number-resolving capability and high dynamic range performance. This is the highest pixel resolution ever reported among low-noise photon-number-resolving image sensors that has been experimentally validated. This QIS was fabricated with a state-of-the-art, commercial CMOS process using 2-layer wafer stacking and BSI, making these sensors suitable for mass production, unlike other specialized single photon detection systems. Its reliable photon-number-resolving capability is achieved due to an average read noise of 0.35 e- rms, putting it well below other scientific imaging systems without sacrificing field of view for increased spatial resolution. This extremely low read noise in conjunction with the DPG readout structure and extended full-well capacity of 20k e- helps to realize a single-exposure dynamic range of 95 dB which is quite competitive and avoids motion artifacts associated with multiple exposure HDR. This superior low-light and HDR imaging performance are all demonstrated with a large imaging array with high spatial resolution. The realization of this detector is another key milestone towards the development of the Gigapixel QIS concept and is an ideal imaging solution to bring ultra-high resolution, large optical format, photon-number-resolving capability and HDR imaging performance to numerous applications including smartphones, industrial imaging, medical diagnostics, scientific and life science imaging. The future generations of this QIS will involve the further improvement of the spatial resolution towards the gigapixel mark and beyond, the further shrinking of the pixel pitch size to below 1 µm, the further optimization of the power consumption, and increased sensor-level integration of advanced image processing circuitry for computational imaging applications.

## Data Availability

The data that support the findings of this study are available from the corresponding authors upon reasonable request.
